# Itinerant-localized dichotomy in magnetic anisotropic properties of U-based ferromagnets

**DOI:** 10.1038/s41598-023-29823-2

**Published:** 2023-02-14

**Authors:** Alexander B. Shick, Itzhak Halevy, Maxim Tchaplianka, Dominik Legut

**Affiliations:** 1 Institute of Physics, Czech Academy of Sciences, Na Slovance 2, 18221 Prague, Czech Republic; 2 Department of Molecular Chemistry and Materials Science, Weizmann Institute of Science, 76100 Rehovoth, Israel; 3 IT4Innovations and Nanotechnology Centre, CEET, VSB-Technical University of Ostrava, 17. listopadu 2172/15, 70800 Ostrava-Poruba, Czech Republic; 4 Nuclear Engineering Unit, Ben Gurion University of the Negev, 84105 Beer-sheva, Israel

**Keywords:** Condensed-matter physics, Materials for energy and catalysis, Theory and computation

## Abstract

The electronic structure, spin and orbital magnetic moments, and the magnetic anisotropy energy in selected U-based compounds are investigated making use of the correlated band theory. First, we demonstrate that the LSDA+U approach with exact atomic limit implemented as a combination of the relativistic density functional theory with the Anderson impurity model provides a good quantitative description for UGa${}_2$. Further, the method is applied to UFe$_{12}$ and UFe${}_{10}$Si${}_2$ ferromagnets. The calculated positive uniaxial magnetic anisotropy together with negative enthalpy of formation for UFe${}_{10}$Si${}_2$ make it as a candidate for the magnetically hard materials. Our studies suggest a viable route for further development of the rare-earth-lean permanent magnets by replacing a part of U atoms by some rare-earth like Sm in UFe${}_{10}$Si${}_2$.

## Introduction

There is a revival of interest in exploiting the electronic and magnetic character of quantum matter closely connected to unconventional superconductivity with non-trivial topology^1^. Only a handful of quantum materials that show coexistence of ferromagnetism and superconductivity are the uranium-based compounds including UGe$_2$^2^, UTe$_2$^3^, URhGe^4^ and UCoGe^5^. The 5*f* electrons in these materials exhibit competing itinerant and localized characteristics between magnetic, and heavy Fermi liquid behaviour.

This itinerant-localized dichotomy is itself not so unusual in metallic actinide compounds, reflecting the “dual” nature^6^ often exhibited by open 5*f* shells. Hill recognized the shortest U–U separation as a critical parameter in uranium intermetallics and provided the “Hill plot”^7,8^ of magnetic or superconducting ordering temperature versus U–U separation. If shorter than 3.5 Å, the 5*f* states are itinerant and sometimes superconducting, if longer they display a local moment and usually order. This Hill’s critical separation is not absolute: it was found to be violated in the heavy fermion metals UPt$_3$ and UBe$_{13}$ found to be superconducting without magnetic order. Still, the Hill criterion is a very useful and physically motivated guide.

This dual nature of the 5*f* shell requires that both local and itinerant features of the *f*-electrons may need to be allowed in a description of the electronic structure of uranium compounds. Experimentally, they can be examined by photoemission spectroscopy (PES) where the localization would lead to a low density of states (DOS) at the Fermi level ($E_F$). Experimental PES can be compared to the results of the density functional theory (DFT) calculations in order to examine validity of DFT, and to explain the experimental data in terms of quantitative and material specific electronic band structure^9^.

The itinerant-localized dichotomy of the actinides plays not only a fundamental role, but can have important practical implications. In the resent years there is an extensive research targeting new permanent magnets with reduced amount of the rare-earth^10^. A good permanent magnet ought to have reasonably high Curie temperature, a large magnetisation for a high energy product, and a substantial uniaxial magnetic anisotropy to resist demagnetisation^11^. In some of the uranium intermetallics^12^ the U-atoms carry large magnetic moments (both spin and orbital), and a huge magnetic anisotropy. Uranium compounds U-(T = 3*d*) with a high content of the transitional metals, in which both U and 3*d* atoms are magnetic, have a potential as magnetically hard materials. Unfortunately, in most of the cases the anisotropy has a multiaxial type (easy-plane)^12^. The only thermodynamically stable U compound with high uniaxial anisotropy, and high Curie temperature of 650 K is UFe${}_{10}$Si${}_2$^13^, and shares the same ThMn$_{12}$-type tetragonal crystal structure as some of the rare-earth RE(Fe,Si)${}_{12}$ compounds^14,15^.

Here, we examine the itinerant-localized dichotomy in selected U-based ferromagnets making use of the local-spin-density plus Coulomb-U approach (LSDA+U). At first, we recall the electronic and magnetic character of well-known UGa$${}_2$$ ferromagnet, and make a comparison between itinerant and localized flavours of LSDA+U. These results are compared with previous experimental and theoretical works and additional features are pointed out. Next, the electronic structure, spin and orbital magnetic moments, and the magnetic anisotropy energy (MAE) for UFe$$_{12}$$ and UFe$${}_{10}$$Si$${}_2$$ ferromagnets are presented. We estimate the thermodynamic stability for these materials in terms of the enthalpy of formation, and illustrate that the Si atom substitution into UFe$$_{12}$$ stabilize the ThMn$$_{12}$$-type crystal structure. Moreover, it is shown that localized Hubbard-I approximation yields the uniaxial MAE of UFe$${}_{10}$$Si$${}_2$$ in reasonable agreement with available experimental data. Finally, We suggest that UFe$${}_{10}$$Si$${}_2$$ ferromagnet is a candidate for magnetically hard material, and predict that (Sm$$_{1-x}$$U$$_{x})$$Fe$${}_{10}$$Si$${}_2$$ can be a promising candidate for the rare-earth-lean permanent magnet.

## Results

The U-atom *f* electrons are mainly responsible for itinerant-localized dichotomy, and the external *spd* electrons make only a discreet contribution to it. Nevertheless, their role can not be disregarded, and we assume that electron interactions in the *s*, *p*, and *d* shells are well approximated in DFT. In this work, the electron correlation effects in 5*f*-manifold are treated making use of two different flavours of local-spin-density plus Coulomb-U approach (LSDA+U): (1) an itinerant orbital polarisation limit (LSDA+U(OP)), and (2) a localized Hubbard-I approximation with the exact atomic limit (LSDA+U(HIA)) described in section “Theoretical methods and computational details”. These correlated band theoretical calculations are performed making use of the relativistic version of the full-potential linearized augmented plane wave (FP-LAPW) method including SOC, combined with the rotationally invariant form of LSDA+U implemented as described in Refs.^16,17^.

### UGa$_2$

UGa$_2$ crystallizes in a hexagonal AlB$_2$ structure^18^ (*P*6/*mmm* space group #191). The Ga atoms separate effectively the U atoms, and the shortest U–U interatomic distance of 4.2 Åis well above the Hill limit^7^. This dictates the formation of the local magnetic moments of 2.7^18^–3.0^19^ $\mu _B$ at the U atoms which are ferromagnetically ordered below the Curie temperature of 125 K.

Experimentally, a large magnetocrystalline anisotropy of − 17 meV/f.u. is found in a single crystal of UGa$_2$^18^ which keeps the magnetization along the [100] direction. The soft-x-ray photoemission (PES) was measured^20^ on a single crystal in a ferromagnetic phase well below the Curie temperature. The spectrum shows a narrow peak below the Fermi energy $E_F$ followed by two broader features at 0.5 and 1 eV binding energies, and a hump at about 2.8 eV. Very recently, an unoccupied electron spectrum was investigated by HERFD-XAS^9^ in a paramagnetic phase.

Theoretically, there were a few investigations of the electronic structure and magnetic character of UGa$_2$ ferromagnet^21,22^ making use of conventional DFT(LSDA/GGA). These theoretical results^21,22^ do not match well the experimental data. Very recently, the electronic and magnetic character of UGa$_2$ were re-examined^9,23^ making use of Wien2k^24^ and FPLO^25^ codes, and carefully compared with available experimental data. Neither LSDA nor GGA with the SOC included reproduce well experimental value of the magnetization, as it is expected for the materials with narrow 5*f* bands and large orbital magnetic moments.

Different flavours of LSDA+U^23^ with Coulomb $U=$ 1, 2 eV, and exchange $J=$ 0.4, 0.6 eV chosen in the ballpark of commonly asserted values for the uranium intermetallic compounds, as well as GGA plus the orbital polarization correction^26^ (GGA+OPC) yield the only partial improvement for the value of the magnetization over DFT results. Also, GGA+OPC reproduce some important features of the experimental PES and XAS spectra, but not to a satisfactory amount.

The magnetic anisotropy energy (MAE) of UGa$_2$ is investigated in Ref.^23^ making use of DFT(LSDA/GGA) and DFT+U. It is evaluated as a difference in the total energes between [100] and [001] magnetization directions, MAE = $E^{[100]} - E^{[001]}$. In contrast to earler LSDA results^21^, they report the negative MAE which corresponds to correct [100] easy axis. However the MAE magnitude is substantially smaller than the experimental value^18^. When LSDA+U is applied, the MAE becomes positive, and the [001] easy axis is incorrect, except of the choice for Coulomb $U=$ 0.8 eV and unphysicaly small exchange $J=$ 0.2 eV values. Simultaneously, the magnetization value is improved over DFT results. The use of spherically symmetric flavour of DFT+U^27^ improves the sign of the MAE but worsens the magnetization value. Note that this version of DFT+U is generally not suitable for the uranium-based materials with strong SOC and/or non-collinear magnets^28^.

Following a conventional approach, we make use of reduced atomic Hartree–Fock values^29^ of the Slater integrals $F_2$ = 6.20 eV, $F_4$ = 4.03 eV, and $F_6$ = 2.94 eV. The resulting values are Hund’s *J* = 0.51 eV, and we select a Hubbard *U* (= $F_0$) equal to the value of *J* for LSDA+U(OP) calculations. With this choice of the Coulomb repulsion *U* equal to the Hund’s exchange *J*, all spherically symmetric terms in the rotationally invariant *U*, *J* correction are set to zero.

In Table 1, we show the occupation of the 5f shell $n_f$, the $m_S$ and $m_L$ in the uranium atom 5f shell, the ratio $-m_{L} / m_{S}$, the total magnetic moment in the unit cell for [100] and [001] directions of the magnetization, and the MAE. Our LSDA results are in good agreement with recently reported WIEN2k calculations^23^. The total magnetic moment per the unit cell is significantly smaller than experimentally reported data^18^. The easy magnetization direction [100] is reproduced correctly, but the magnitude of the MAE is about a half of the experimental value. When the LSDA+U(OP) is used, the total magnetic moment values are improved, mainly due to an enhancement of the U 5f-shell orbital moment $m_L$, but the MAE becomes positive with the wrong [001] easy axis.

Next, we consider the localized 5f-shell model making use of the LSDA+U(HIA) approximation. The Coulomb repulsion *U* = 3 eV is used together with Hund’s *J* = 0.51 eV^30^. The results are shown in Table 1. The magnitude of the U 5f-shell orbital moment $m_L$ is strongly enhanced with respect to LSDA and LSDA+U(OP). The total magnetic moment in the unit cell agrees well with the experimental value of 2.7 $\mu _B$^18^ obtained from the magnetization measurements, and the U-atom 5f-shell moment of 2.9 $\mu _B$ agrees well with the magnetic moment of 3.0 ± 0.1 $\mu _B$^19^ from polarized neutron scattering data. The negative MAE of − 18.98 meV per unit cell is evaluated in a good quantitative agreement with the experimental value. The minus sign of MAE means that the [100] is the easy magnetization axis in agreement with experimental observation.

**Figure 1:**
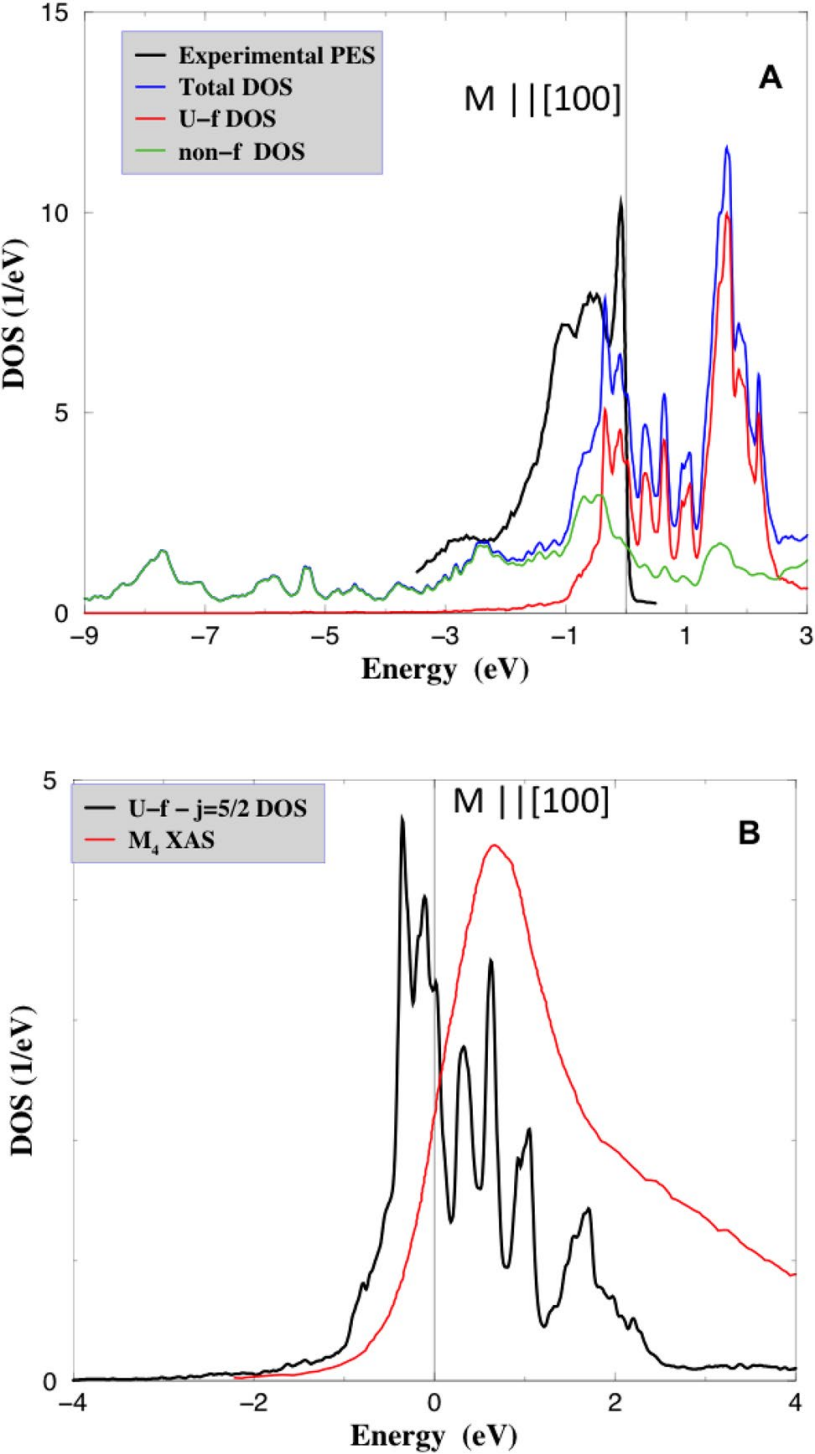
UGa$_2$—(**A**) Experimental photoelectron spectra (black line) from^20^ are compared to the total DOS, orbital-resolved U-5f and a sum of all non-f DOS. (**B**) Experimental HERFD XAS spectra for U-5f (black line) from^9^ are compared to the calculated $j=5/2$-projected U-f DOS. All theoretical DOS correspond to the ferromagnetic solution with [100] magnetic moment alignments.

In Fig. 1A, we show the total DOS, the U-5f projected DOS and a sum of all non-f DOS for ferromagnetic UGa$_2$ with the magnetization along the [100] easy direction, in a comparison with the experimental PES^20^. There is a narrow peak in the DOS $\approx$ 0.1 eV below $E_F$, in agreement with PES which has the 5f-character. Next to it there is another sharp peak in the DOS at $\approx$ 0.35 eV binding energy with the 5f-character, and a broader feature between 0.4 and 0.7 eV of the U-6d and Ga-4p non-f character. Experimentally, there are two relatively broad features at 0.5 and 1.0 eV. This inconsistency between the theoretical DOS and the experimental PES comes from a luck of hybridization between Ga-4p and U-5f states in the Hubbard-I approximation. When the exact diagonalization (Lanczos) is used^31^ instead, agreement between theory and experiment is somewhat improved.

The unoccupied part of the spectrum can be accessed by an X-ray adsorption spectroscopy making use of a high-energy resolution fluorescence detection mode (HERFD-XAS) at the uranium $M_4$ edge^9^. In Fig. 1B, the experimental $M_4$ spectra are compared with the projected $j=5/2$ U-5f DOS (the experimental spectrum is aligned with the theoretical main peak above the $E_F$). It is seen that $j=5/2$ f-DOS is in a reasonable agreement with the experimental data as well as the LSDA+DMFT results^9^.

Finally, our calculations indicate that the itinerant LSDA and LSDA+U(OP) treatment of the U 5f-manifold does not provide the consistent description of magnetism in UGa$_2$. The LSDA+U(HIA) calculations illustrate that the local Hubbard-I model with exact atomic limit for treating 5f-electrons in UGa$_2$ describes the magnetic, anisotropic and spectroscopic properties better than the conventional DFT or DFT+OPC, in a reasonable quantitative agreement with more sophisticated and computationally demanding LSDA+DMFT^31^.

**Table 1:** UGa$_2$—the occupation of the 5f shell $n_f$, the spin and orbital magnetic moments in the uranium atom 5f shell, $m_S$ and $m_L$ (in $\mu _B$), their ratio $-m_{L} / m_{S}$, the total magnetic moment in the unit cell $m_{tot}$ (in $\mu _B$), and the magnetic anisotropy energy MAE (in meV) in comparison with experimental data.

	dir.	$n_f$	$m_{S}$	$m_{L}$	-$\frac{m_{L}}{m_{S}}$	$m_{tot}$	MAE
LSDA	[100]	2.66	− 1.93	2.76	1.47	0.61	
LSDA	[001]	2.66	− 1.98	2.79	1.41	0.55	− 11.90
LSDA+U(OP)	[100]	2.73	− 1.63	3.73	2.29	1.86	
LSDA+U(OP)	[001]	2.73	− 1.60	3.47	2.17	1.69	11.81
LSDA+U(HIA)	[100]	2.71	− 1.83	4.76	2.93	2.71	
LSDA+U(HIA)	[001]	2.75	− 1.83	4.75	2.60	2.70	− 18.98
Exp.					1.9–3.0	3.0 ± 0.1	− 17.1

The results of LSDA, LSDA+U(OP), and LSDA+U(HIA) for the magnetization constrained along [100] and [001] crystal directions are shown.

### UFe$_{12}$ and UFe${}_{10}$Si${}_2$

**Figure 2:**
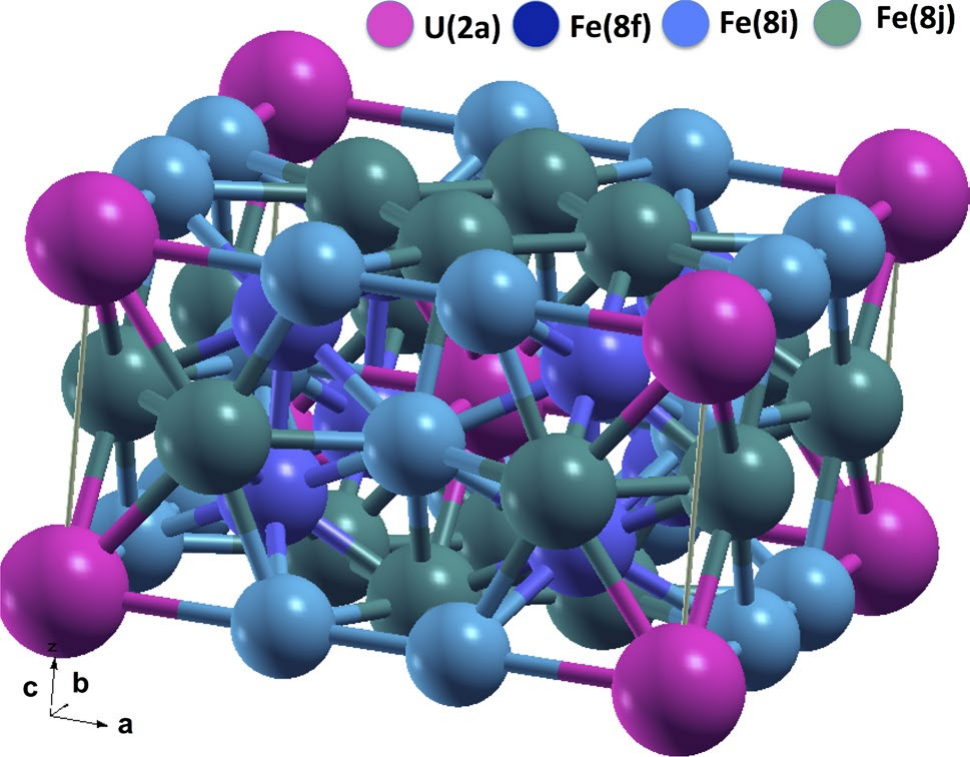
The ThMn$_{12}$-type lattice of UFe${}_{12}$ with uranium atoms in 2a Wyckoff position shown in pink, and the Fe atoms in 8f (dark blue), 8j (light blue), and 8i (green) Wyckoff positions. The uranium and iron magnetic moments are aligned along the [001] direction.

Next, we turn to salient aspect of our investigation, the magnetic anisotropic properties of UFe$_{12}$ and UFe${}_{10}$Si${}_2$. The body-centered tetragonal ThMn$_{12}$-type crystal structure (I4/mmm) is shown in Fig. 2. In the calculations, we use the experimental crystal and internal parameters^32^. The FP-LAPW basis parameters, and values of Slater integrals are chosen as for the case of UGa$_2$.

The spin $m_S$ and orbital $m_L$ magnetic moments (in $\mu _B$) aligned along [001] crystal direction for U and Fe atoms in different Wyckoff positions, calculated with LSDA+U(HIA) are shown in Table 2. We note the anti-parallel alignment of the U and Fe spin $m_S$ magnetic moments in accordance to well established mechanism^33,34^ in the rare-earth-Fe intermetallic compounds. Comparison with the results of LSDA+U(OP) (see Table S1 in the Supplemental information) shows that the main difference comes from enhancement of the orbital magnetic moment on the U-atom in LSDA+U(HIA) , while both the spin and orbital moments of the Fe atoms remain almost untouched by the 5*f*-shell Coulomb interactions.

**Figure 3:**
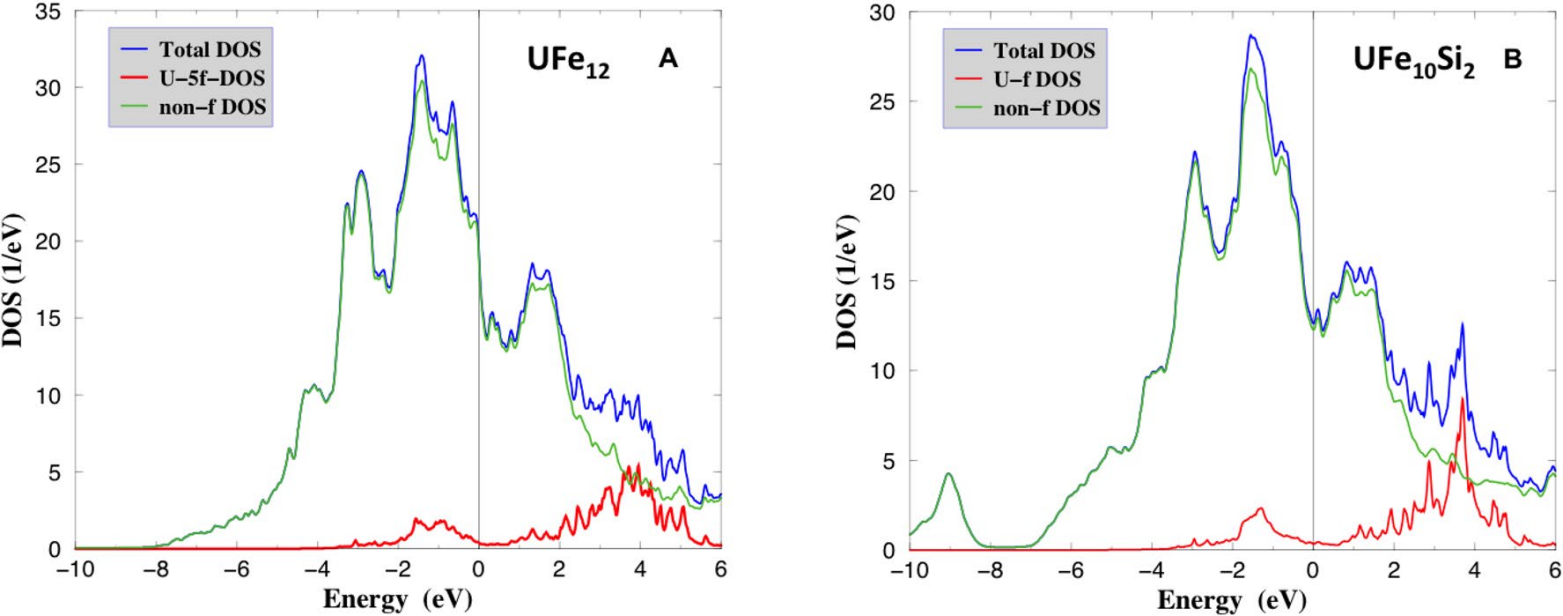
(**A**) The total DOS, orbital-resolved U-5f and a sum of all non-f DOS for UFe${}_{12}$. (**B**) The total DOS, orbital-resolved U-5f and a sum of all non-f DOS for UFe${}_{10}$Si${}_2$.

In Fig. 3A, we show the total DOS, the U-5f projected DOS and a sum of all non-f DOS for ferromagnetic UFe${}_{12}$ with the magnetization along the [001] easy axis as a result of LSDA+U(HIA) calculations. It is seen that most of the total DOS near $E_F$ does not have the *f*-character, and stems from the Fe atom *d*-states. The *f*-states are located $\approx$ 0.5 to 1.5 eV below the Fermi edge. On a contrary, in the itinerant LSDA+U(OP) picture the total DOS (see Fig. S1 in the Supplemental information) includes the U-5f DOS contribution at $E_F$. As it is expected, the *f*-states are substantially more delocalized.

The LSDA+U(OP) yields the strong and negative MAE = − 67.5 meV/f.u., which corresponds to the easy-plane magnetic moment orientation along the [100] axis. The LSDA+U(HIA) calculated MAE = 11.12 meV/f.u. is positive, and is comprised from the U-atom f-shell contribution (8.9 meV), and the Fe contribution of 2.2 meV/f.u. Thus, the localized model predicts the uniaxial MAE for UFe$_{12}$.

We estimate the structural stability of UFe${}_{12}$ calculating the enthalpy of formation $\Delta H$, $\Delta H=E-\sum _{i}\mu _i x_i$, where *E* is the LSDA total energy of the UFe${}_{12}$, $\mu _i$ is the chemical potential of element *i* and $x_i$ is the quantity of element *i* in the compound^35^. The standard convention is to take the chemical potential of each species to be the DFT total energy of the elemental Fe and $\alpha$-U ground state. The enthalpy of formation is found to be $\Delta H$ = 3.349 eV/f.u. The positive sign of $\Delta H$ indicates that pristine UFe${}_{12}$ is not thermodynamicaly stable.

**Table 2:** The spin $m_S$ and orbital $m_L$ magnetic moments (in $\mu _B$) for U and Fe atoms in different Wyckoff positions, aligned along [001] crystal direction.

Atom	U (2a)		Fe (8f)	Fe (8i)	Fe (8j)	Unit cell
UFe${}_{12}$						
$m_{S}$	− 2.28		1.68	2.42	2.05	21.94
$m_{L}$	4.57		0.06	0.10	0.09	5.61
$m_{tot}$	2.29		1.74	2.52	2.14	27.55
U-atom MAE = 8.92 meV				Total MAE = 11.12 meV		

Atom	U (2a)	Si(8f)	Fe (8f)	Fe (8i)	Fe (8j)	Unit cell
UFe${}_{10}$Si${}_2$						
$m_{S}$	− 2.28	− 0.08	1.69	2.29	1.91	17.25
$m_{L}$	4.70	0.00	0.07	0.11	0.11	5.73
$m_{tot}$	2.42	− 0.08	1.76	2.40	2.02	21.92
U-atom MAE = 2.56 meV				Total MAE = 3.99 meV		
Exp. MAE = 3.1 meV (3 MJ/m$^3$), $m_{tot}$ = 16.5 $\mu _B$						

The total magnetic moment in the unit cell $m_{tot}$, and the magnetic anisotropy energy MAE (in meV) in comparison with experimental data.

The available experimental data^13–15^ confirm stability of the tetragonal ThMn$_{12}$-type structure for some rare earth and uranium compounds RFe${}_{10}$Si${}_2$, when the rare earth or actinide R have an atomic radius smaller than 1.81 Å^36^, and Fe is substituted by Si. We consider the effect of Si substitution in UFe${}_{10}$Si${}_2$. Experimentally, the Si atoms randomly occupy the (8f) and (8j) Wyckoff positions of the Fe-atoms^13,32^ shown in Fig. 2. This inherent disorder can not be modeled in our calculations. In order to discern the electronic structure of UFe${}_{10}$Si${}_2$ we assume that Si atoms occupy the (8f) sites only, keep the thirteen atoms unit cell, and preserve the inversion symmetry (see Fig. S2 in the Supplemental information). At first, we estimate the enthalpy of formation $\Delta H$ = − 1.425 eV/f.u. in LSDA. Negative sign of $\Delta H$ points on the thermodynamic stability of UFe${}_{10}$Si${}_2$ upon the Fe atom replacement by Si, in qualitative agreement with available experiment.

We cannot estimate the enthalpy of formation $\Delta H$ within the localized LSDA+U(HIA) model since the different total energy functionals are used for UFe${}_{12}$ and UFe${}_{10}$Si${}_2$ on the one hand, and $\alpha$-U on the other hand. Nevertheless, we can estimate the change of the enthalpy due to the Fe atom substitution by the Si atom, and have found that $\Delta H$(UFe${}_{10}$Si${}_2) - \Delta H$( UFe${}_{12}$) = − 3.234 eV. It means that the Si substitution tends to thermodynamically stabilise the crystal structure. Note that this comparison has to be taken with some cautiousness since it is done for *T* = 0 K and without phonon contribution.

The spin $m_S$ and orbital $m_L$ magnetic moments (in $\mu _B$) aligned along [001] crystal direction for U, Si and Fe atoms in different Wyckoff positions, calculated with LSDA+U(HIA) are shown in Table 2. We note that the U-atom spin and orbital magnetic moments are weakly affected by Si substitution. The Si atoms remain almost non-magnetic. While the Fe atoms in (8f) Wyckoff positions keep almost the same spin and orbital magnetic character as in UFe${}_{12}$, the $m_S$ on Fe-(8j), and especially on Fe-(8i) is reduced. The resulting total magnetic moment per unit cell of 21.9 $\mu _B$ is substantially reduced from the 27.6 $\mu _B$ value for UFe${}_{12}$, bringing it somewhat closer to the experimental 16.5 $\mu _B$ value.

In Fig. 3B, we show the total DOS, the U-5f projected DOS and a sum of all non-f DOS for ferromagnetic UFe${}_{10}$Si${}_2$ with the magnetization along the [001] easy axis as a result of LSDA+U(HIA) calculations. Similar to the case of UFe${}_{12}$ (cf. Fig. 3B) most of the total DOS near $E_F$ does not have the *f*-character. Again, the Fe atom *d*-states contribute the most into the total DOS, which is reduced in a comparison with UFe${}_{12}$ following a reduction of a number of the Fe atoms. The *f*-states are peaked at $\approx$ 1.5 eV below the Fermi edge. On a contrary, in the itinerant LSDA+U(OP) picture, the total DOS (see Fig. S1B in the Supplemental information) includes the U-5f DOS contribution at $E_F$, and the U-5*f*-states are substantially more delocalized in LSDA+U(OP) calculations.

The LSDA+U(HIA) calculated MAE = 4.0 meV/f.u. is positive, and consists of the U-atom single-ion contribution (2.6 meV), and the Fe contribution of 1.4 meV/f.u. Thus, the localized LSDA+U(HIA) model predicts the uniaxial MAE for UFe${}_{10}$Si${}_2$ in a reasonable quantitative agreement with the experimental value of 3.1 meV/f.u.^13^.

## Discussion and summary

To be a good magnet, a ferromagnetic compound should have a Curie temperature, $T_c$, above 400 K, a saturation moment in a range of 1 MA/m, and the uniaxial magnetic anisotropy of about 4 MJ/m$^3$^37^. For instance, one of the best permanent magnets, Nd${}_2$Fe${}_{14}$B, has a $T_c$ of 588 K, the saturation moment of 1.28 MA/m, and the uniaxial MAE of 4.9 MJ/m$^3$ with the tetragonal easy *c*-axis^38^.

As follows from Table 2 for UFe${}_{10}$Si${}_2$, the calculated magnetic moment is of 1.23 MA/m ($\mu _0 M_s$ = 1.55 T), and the MAE density, $K_u$ = 2.41 MJ/m$^3$, is somewhat below the standard ($\approx$ 4 MJ/m$^3$) required for good permanent magnets^11^. The anisotropy field estimated as $H_a = 2 K_u / M_s$ = 3.9 T which is slightly above the required value of 3.75 T^39^. The maximum energy product $BH_{max} = {1/4} \mu _0 {M_s}^2$ = 475 KJ/m$^3$ is comparable to $BH_{max} \approx$ 460 KJ/m$^3$ in the rare-earth intermetallics Nd${}_{2}$Fe${}_{14}$B^40^. The hardness parameter^41^ $k = \sqrt{K_u / \mu _0 {M_s}^2}$ = 1.13. Thus, UFe${}_{10}$Si${}_2$ can be considered as a candidate to the magnetically hard material. We note that LSDA+U(HIA) calculations (as well as LSDA+U(OP) shown in Supplemental information Table S1) overestimate the total magnetic moment for UFe${}_{10}$Si${}_2$ by $\approx$ 25%. Once we take this difference into account, the anisotropy field $H_a$ = 5.2 T will be increased, and the energy product $BH_{max}$ = 267 KJ/m$^3$ will be decreased.

The SmFe$${}_{12}$$ family has long been of interest as the rare-earth-lean hard magnetic materials^42^. Replacing the Sm atoms with atomic radius of 1.81 Å by smaller Zr atoms (1.60 Å)^43^ leads to stabilisation of (Sm$$_{0.7}$$Zr$$_{0.3})$$Fe$${}_{10}$$Si$${}_2$$ alloys into the 1:12 ThMn$$_{12}$$-type structure^44^. Since the empirical atomic radius of uranium (1.56 Å) is close to Zr, we expect the (Sm$$_{1-x}$$U$$_{x})$$Fe$${}_{10}$$Si$${}_2$$ alloys to crystallize in 1:12 structure. Moreover, since experimental $$K_u$$ for SmFe$${}_{10}$$Si$${}_2$$ is $$\approx$$ 7 to 8 MJ/m$$^3$$^45^, the (Sm$$_{1-x}$$U$$_{x})$$Fe$${}_{10}$$Si$${}_2$$ alloy is expected to have a higher $$K_u$$ than UFe$${}_{10}$$Si$${}_2$$. We note that since the $$T_c$$ for UFe$${}_{10}$$Si$${}_2$$ is about 640 K^46^, and about 600 K^45^ for SmFe$${}_{10}$$Si$${}_2$$, the (Sm$$_{1-x}$$U$$_{x})$$Fe$${}_{10}$$Si$${}_2$$ alloy is expected to have reasonably high $$T_c$$ above 400 K. Since LSDA+U(HIA) can achieve an accurate description of the Sm and U atoms *f*-shell, it can serve in a future investigation of magnetism and magnetic anisotropy in (Sm$$_{1-x}$$U$$_{x})$$Fe$${}_{10}$$Si$${}_2$$.

It is to note that while values of $T_c$, $M_s$, $K_u$ determine the ultimate limits for the permanent magnet, they are necessary but not sufficient. Besides the intrinsic bulk properties it is necessary to have a material in which the microstructure can be controlled to yield high coercivity; this is limited by $K_u$ but depends in a complicated way on the microstructure at the mesoscale. Therefore, while good intrinsic properties are essential, one also seeks a ferromagnet that is readily synthesized and amenable to processing.

In summary, our correlated band theory LSDA+U(HIA) calculations, motivated by the Anderson impurity model suggest that the multiconfigurational aspect of the U 5*f*-shell together with a correct atomic limit need to be taken into account in order to reproduce the anisotropic magnetic and spectroscopic properties of selected U-based ferromagnets. Exploration of the itinerant-delocalised dichotomy in UGa${}_2$ demonstrates that U 5*f*-shell is well localized, and LSDA+U(HIA) calculations reproduce reasonably well the spin, orbital and the total magnetic moments, and the MAE together with occupied and unoccupied electronic structure.

For UFe$$_{12}$$ and UFe$${}_{10}$$Si$${}_2$$ ferromagnets we explored the thermodynamic stability in terms of the enthalpy of formation, and found out that the Fe atom substitution by the Si atom tends to stabilise the ThMn$$_{12}$$-type crystal structure. Furthermore, our LSDA+U(HIA) calculations suggest that UFe$${}_{10}$$Si$${}_2$$ ferromagnet is close to fulfil criteria for magnetically hard material. These results suggest a viable route for enhancing structural stability and intrinsic hard magnetic properties of UFe$${}_{10}$$Si$${}_2$$ by replacing a part of U atoms by some rare-earth. We predict that (Sm$$_{1-x}$$U$$_{x})$$Fe$${}_{10}$$Si$${}_2$$ can be a promising candidate predict that (Sm$$_{1-x}$$U$$_{x})$$Fe$${}_{10}$$Si$${}_2$$ can be a promising candidate for the magnetically hard material. Our studies can have an important impact on further development of the rare-earth-lean permanent magnets.

## Theoretical method and computational details

Theoretical calculations are preformed using the LSDA+U total energy functional in the rotationally invariant form with the spin-orbit coupling (SOC) included^47,48^,

1 $$\begin{aligned} E^{ee} = \frac{1}{2} \sum _\mathbf{\gamma _1 \gamma _2 \gamma _3 \gamma _4} n_\mathbf{\gamma _1 \gamma _2} \Big ( V^{ee}_\mathbf{\gamma _1 \gamma _3; \gamma _2 \gamma _4} - V^{ee}_\mathbf{\gamma _1 \gamma _3;\gamma _4 \gamma _2} \Big ) n_\mathbf{\gamma _3 \gamma _4} \;, \end{aligned}$$

which contains the $14\times 14$ on-site occupation matrix $n_\mathbf{\gamma _1 \gamma _2} \equiv n_{m_1 \sigma _1,m_2 \sigma _2}$ with generally non-zero orbital and spin off-diagonal matrix elements. The $V^{ee}$ is an effective on-site Coulomb interaction, expressed in terms of Slater integrals^16^. The spherically symmetric double-counting energy $E^{dc}$ is subtracted from $E^{ee}$ to correct on the electron-electron interaction already included in DFT. An additional non-spherical double-counting correction is used as described in Ref.^49^.

Two flavours of LSDA+U are used. In the first approach, the LSDA+U energy correction $\Delta E^{ee} = E^{ee} - E^{dc}$ can be divided into a sum of spherically symmetric^27^ and anisotropic terms. With the artificial choice $U = J$ in Eq. (1), the spherically symmetric part of $\Delta E^{ee}$ becomes equal to zero. The remaining anisotropic part of $\Delta E^{ee}$ can be regarded as analog of “orbital polarization correction”^26,50^ implemented in the LSDA+U language. Therefore, we call it LSDA+U(OP)^51,52^, and regard it as an *itinerant model* due to a small value of Coulomb-U which enters the calculations.

In LSDA+U(OP), the MAE is evaluated as a difference in the total energes between [100] and [001] magnetization directions, MAE = $E^{[100]} - E^{[001]}$. Note that the magnetic anisotropy calculation is an involved endeavor, and requires a special care due to a need to insulate small energy differences from a total energy which may be many orders of magnitude larger^53^. The same k-points mesh in the Brillouin zone (BZ) is used in the calculations for both magnetization directions. The special k-points method is used for the BZ integration with the Gaussian smearing of 1 mRy for k-points weighting. The total energy convergence better than 0.01 meV is preserved in the self-consistent LSDA+U(OP) calculations. In order to achieve convergence of the MAE with desired accuracy, about 10,000 k-points are used.

Next is a localized approach^30,54^, where it is assumed that the hybridization between the localized *f*-electrons and the itinerant *s*, *p*, and *d*-states is weak, so that the *f*-shell is described with the aid of seven-orbital *f*-shell Anderson impurity model^55^ reduced to the atomic limit, also known as the Hubbard-I approximation (HIA)

2 $$\begin{aligned} H_{\textrm{imp}} =&\sum _{m\sigma } \varepsilon _f f^{\dagger }_{m \sigma }f_{m \sigma } + \sum _{mm'\sigma \sigma '} \bigl (\xi \textbf{l}\cdot \textbf{s} + \Delta _{\textrm{CF}} + \frac{\Delta _{\textrm{EX}}}{2} {\hat{\sigma }}_z\bigr )_{m\sigma ,m'\sigma '} f_{m \sigma }^{\dagger }f_{m' \sigma '} \\&+ \frac{1}{2} \sum _{\begin{array}{c} m m' m''\\ m''' \sigma \sigma ' \end{array}} U_{m m' m'' m'''} f^{\dagger }_{m\sigma } f^{\dagger }_{m' \sigma '} f_{m'''\sigma '} f_{m'' \sigma }. \nonumber \end{aligned}$$

where $f^{\dagger }_{m \sigma }$ creates an electron in the *f* shell. The parameter $\xi$ specifies the strength of the SOC, $\Delta _{\textrm{CF}}$ is the crystal-field potential at the impurity, and $\Delta _{\textrm{EX}}$ is the strength of the exchange field. The energy position $\varepsilon _f$ ($= -\mu$, the chemical potential) defines the number of *f*-electrons. All of these parameters are calculated self-consistently. The last term describes the Coulomb interactions in the *f*-shell.

The Lanczos method^56^ is employed to find the eigenstates of the many-body Hamiltonian $H_{\textrm{imp}}$ Eq. (2) and to calculate the one-particle Green’s function $[G_{\textrm{imp}}(z)]_{m m'}^{\sigma \; \; \sigma '}$ in the subspace of the *f* orbitals. The self-energy $[\Sigma (z)]_{m m'}^{\sigma \; \; \sigma '}$ is then obtained from the inverse of the Green’s-function matrix $[G_{\textrm{imp}}]$. Once the self-energy is known, the local Green’s function *G*(*z*) for the electrons in the solid,

3 $$\begin{aligned}{}[G(z)]_{\gamma _1 \gamma _2} = \frac{1}{V_{\textrm{BZ}}} \int _{\textrm{BZ}}\textrm{d}^3 k \,\bigl [z+\mu -H_{\textrm{LDA}}(\textbf{k})-\Sigma (z)\bigr ]^{-1}_{\gamma _1 \gamma _2}, \end{aligned}$$

is calculated in a single-site approximation. The local Green’s function *G*(*z*) is used to evaluate the occupation matrix $n_{\gamma _1 \gamma _2} = -\frac{1}{\pi }\,\mathop {\textrm{Im}} \int _{-\infty }^{E_{\mathrm{{F}}}} \textrm{d} z \, [G(z)]_{\gamma _1 \gamma _2}$ in the LSDA+U total energy functional Eq. (1). Note that in this LSDA+U(HIA) approach the atomic limit is explicitly ensured. Interested reader can find the further details of our implementation in Ref.^30^.

In LSDA+U(HIA) calculations, we used the two-step procedure to estimate the MAE. At first, we evaluate the U-atom single ion anisotropy making use of recipes of the crystal-field (CF) theory^40^. The CF hamiltonian is constructed from the self-consistent LSDA+U(HIA) results^54,57^,

$$\begin{aligned} {\hat{H}}_{CF}=\sum _{kq} A_k^q\langle r^k\rangle \Theta _k(J) {\hat{O}}_k^q \;, \end{aligned}$$

where ${\hat{O}}_k^q$ are the Stevens operator equivalents, $\Theta _k(J)$ are the Stevens factors for a given ground state multiplet *J*, and $A_k^q\langle r^k\rangle$, the crystal field parameters for given *k* and *q*. Next, we follow the CF theory^40^, and identify the magnetic anisotropy ${E}_{MA}(\theta ,\phi )$ as a diagonal element ${\langle {J, J_z=-J }|} {\hat{H}}_{CF} {|{J,J_z=-J}\rangle }$, where the polar ${(\theta ,\phi )}$ angles specify the magnetization direction. The U atom single-ion contribution in uniaxial MAE =[${E}_{MA}(\theta = {\pi /2}) - {E}_{MA}(\theta =0)$] is calculated.

In order to calculate contribution to the MAE from Fe-sublattice in UFe${}_{12}$ and UFe${}_{10}$Si${}_2$, we substituted the uranium atom by the yttrium atom, and performed the LSDA calculations. The magnetic force theorem^58^ together with the magnetic torque approach^59^ were used to evaluate the Fe-sublattice contribution to the uniaxial MAE. This contribution was added to the single-ion U-atom MAE in order to obtain the total anisotropy per unit cell.

## Supplementary Information

Supplementary Information.

## Data Availability

The data used during the current study available from the corresponding author on reasonable request.
