# Supplementary material for: Itinerant-localized dichotomy in magnetic anisotropic properties of U-based ferromagnets
Source: Sci Rep. 2023 Feb 14;13:2646. doi: 10.1038/s41598-023-29823-2 (PMC9929282; doi:10.1038/s41598-023-29823-2)
Supplement: Supplementary file 1 — Supplementary Information. [file 41598_2023_29823_MOESM1_ESM.pdf]

Supplemental Material for
Itinerant-localized dichotomy in magnetic anisotropic properties of U-based ferromagnets

LSDA+U(OP) FOR UFe_{12} AND $UFe_{10}Si_2$

TABLE S1: The spin m_S and orbital m_L magnetic moments (in μ_B) for U and Fe atoms in different Wyckoff positions, and the total magnetic moment in the unit cell m_{tot} aligned along [001] crystal direction as results of LSDA+U(OP) for UFe_{12} and $UFe_{10}Si_2$.

			UFe_{12}			
Atom	U (2a)	Fe (8f)	Fe (8i)	Fe (8j)	Unit cell	
m_S	-1.98	1.69	2.40	2.00	21.97	
m_L	2.30	0.05	0.10	0.09	3.25	
m_{tot}	0.32	1.74	2.50	2.09	25.21	
			$UFe_{10}Si_2$			
Atom	U (2a)	Si(8f)	Fe (8f)	Fe (8i)	Fe (8j)	Unit cell
m_S	-2.76	-0.08	1.72	2.30	1.93	16.98
m_L	2.95	0.00	0.08	0.11	0.11	3.95
m_{tot}	0.19	-0.08	1.80	2.41	2.04	20.93

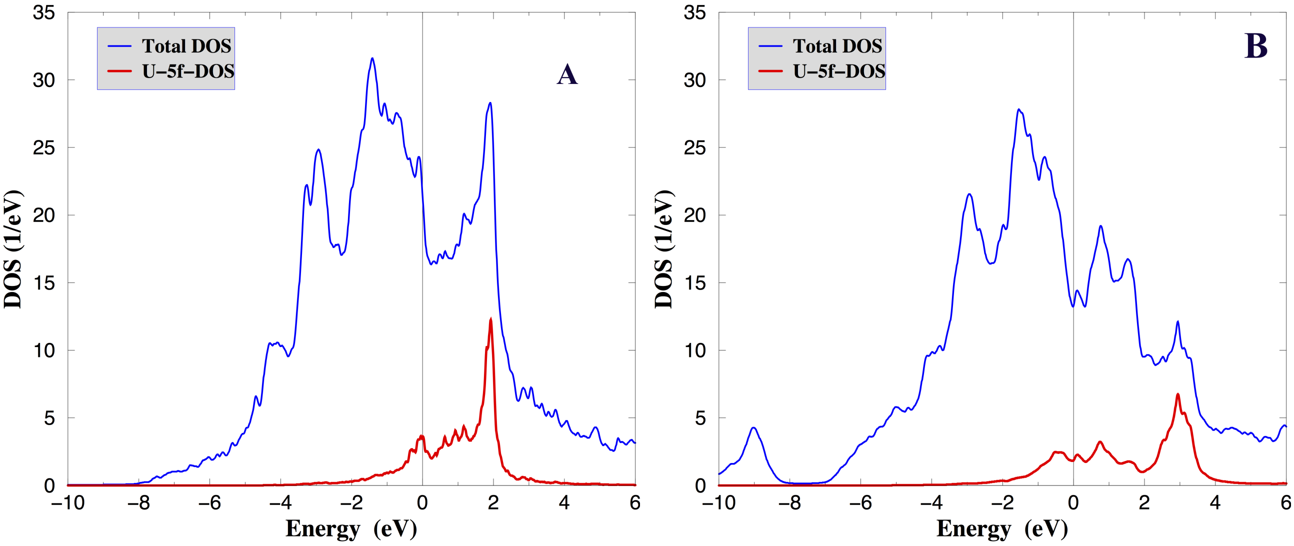

FIG. S1: (A) The total DOS, and U-5f projected DOS for UFe_{12}. (B) The total DOS, and U-5f projected DOS for $UFe_{10}Si_2$

.

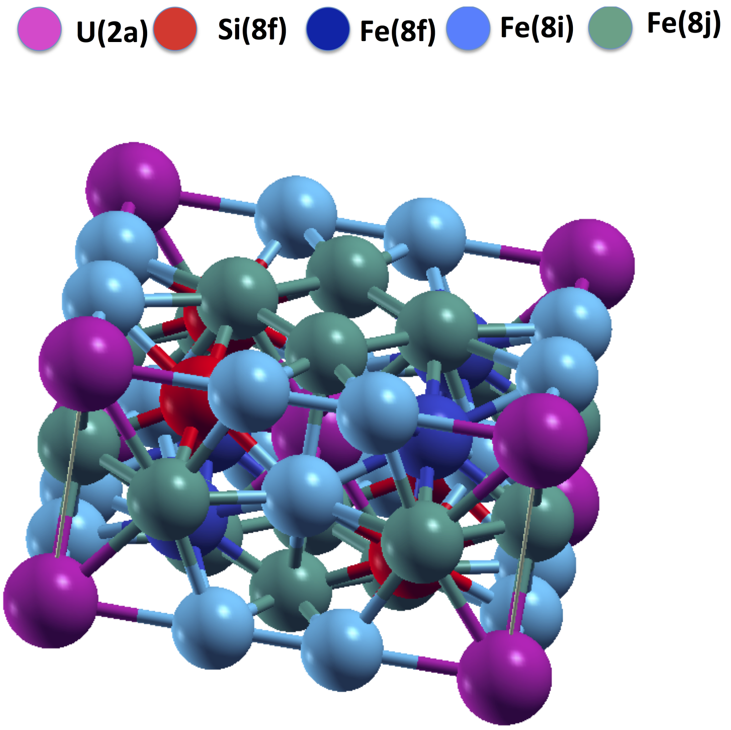

FIG. S2: The $ThMn_{12}$-type lattice of $UFe_{10}Si_2$ with uranium atoms in 2a (pink), silicon atoms in 8f (red), Fe atoms in 8f (dark blue), 8j (light blue), and 8i (green) Wyckoff positions. The uranium and iron magnetic moments are aligned along the [001] direction.
